# Fractional-calculus diffusion equation

**DOI:** 10.1186/1753-4631-4-3

**Published:** 2010-05-21

**Authors:** Abdul-Wali MS Ajlouni, Hussam A Al-Rabai'ah

**Affiliations:** 1 Applied Physics Department, Tafila Technical University, P.O. Box: 179 66110 Tafila- Jordan; 2 Mathematics Department, Tafila Technical University, P.O. Box: 179 66110 Tafila- Jordan

## Abstract

**Background:**

Sequel to the work on the quantization of nonconservative systems using fractional calculus and quantization of a system with Brownian motion, which aims to consider the dissipation effects in quantum-mechanical description of microscale systems.

**Results:**

The canonical quantization of a system represented classically by one-dimensional Fick's law, and the diffusion equation is carried out according to the Dirac method. A suitable Lagrangian, and Hamiltonian, describing the diffusive system, are constructed and the Hamiltonian is transformed to Schrodinger's equation which is solved. An application regarding implementation of the developed mathematical method to the analysis of diffusion, osmosis, which is a biological application of the diffusion process, is carried out. Schrödinger's equation is solved.

**Conclusions:**

The plot of the probability function represents clearly the dissipative and drift forces and hence the osmosis, which agrees totally with the macro-scale view, or the classical-version osmosis.

## Introduction

In this paper we aim to consider the dissipation effects, appeared in the will-known diffusion process, quantum-mechanically depending on the procedure of the quantization of nonconservative systems using fractional calculus [1-4], which was also applied on the related phenomenon, the Brownian motion [5].

Most of the natural laws of physics, such as Maxwell's equations, Newton's laws of motion, and Schrödinger equation, are stated, or can be, in terms of partial deffirential equations (PDEs), that is, these laws describe physical phenomena by relating space and time derivatives. Diffusion equation, or heat flow, is one of the most important PDEs in physical sciences. The basic process in the diffusion phenomenon is the flow of the fluid from a region of higher density to one of lower density [6].

The tendency of a statistical ensemble to achieve thermodynamic equilibrium with a uniform distribution of states for its constituent subsystems does not have to be monotonic in time. In general, equilibration takes place in stages and is characterized by several stochastization times with vastly different orders of magnitude. Thermodynamic equilibrium has no absolute meaning and depends on the time scale over which a given process is analyzed.

In a diffusion process or chemical reaction, Fick's law provides a linear relationship between the flux of molecules and the chemical potential difference. Likewise, a direct proportionality exists between the heat flux and the temperature difference in a thermally conducting slab, as expressed by Fourier's law. Diffusion of gases between air in the lungs and blood proceeds in the direction from high to low concentration, and the rate of diffusion is greatest when the difference in concentration is greatest. Diffusion obeys Fick's law, but the actual rate of exchange is greatly affected by hemoglobin in the blood.

## Diffusion equation

Diffusion is macroscopically associated with a gradient of concentration. In contrast to the mass flow of liquids, diffusion involves random spontaneous movements of individual molecules. The diffusion flux is expressed in number of particles traversing a unit area per unit time and the concentration in number of particles per unit volume. This process can be quantified by a constant known as the *diffusion coefficient*, *D*, of the material, given in general by the *Stokes-Einstein equation*:(1)

where *k*_*B *_is the Boltzmann constant, *T *is the absolute temperature in K, and *f *is a frictional coefficient. The diffusion coefficient, D, is defined as the net flow of particles per unit time across an imaginary plane of unit area lying at right angles to the concentration gradient, that gradient also having unit strength.

Hydrodynamic properties of macromolecules like diffusion, viscosity and sedimentation are affected by the frictional forces between molecules of the diffused material and those of the ambient material. Since this frictional force is in opposition to motion we can include this in the equation of motion as [7]:(2)

where *f *is the frictional coefficient, (*dv/dt*) is the acceleration and m is the mass of the molecule. In the case of spherical particles, the translational frictional force *f *is proportional to the fluid viscosity,η, and radius r of the particle. Thus the coefficient of friction for spherical particles, known as Stokes law, is(3)

The frictional coefficient *f *comes into effect when a molecule moves through a medium. The movement of the molecule could be either diffusion or sedimentation and the driving force, F, can be the concentration gradient, the force of gravity or the centrifugal force. According to Fick's law, the rate of diffusion across a boundary (*dn/dt*: the number of molecules which pass through a cross section *A *in unit time) for a single solute component diffusing in a system at constant temperature and pressure is given by [7]:(4)

where  is the concentration gradient. A concentration gradient implies that the concentration of the molecules (i.e. the solute) varies with distance *r *in one dimension.

1-The continuity equation. The equation of continuity assures us that flow is equal at any point, whatever the degree of tapering. If the cross-sections and corresponding velocities at two points are, respectively, *A*_1_, *A*_2_, *v*_1_, and *v*_2_, from the equation of continuity, we have:(5)

This is a simplified version of the one-dimensional continuity equation whose differential form is(6)

2-Fick's law. Fick's law states that the rate of diffusion per unit area in a direction perpendicular to the area is proportional to the gradient of concentration of solute in that direction. The concentration is the mass of solute per unit volume, and the gradient of concentration is the change in concentration per unit distance. If the concentration changes from *C*_1 _to a lower value of *C*_2 _over a short length (*d*), then the mass (*m*) of the solute diffusing down the pipe in time (*t*) is(7)

This is a simplified version of Fick's law whose differential form is(8)

3- The diffusion equation. In cells without sources, the diffusion equation is written as:(9)

The concentration gradient across the boundary is given as [6]:(10)

where C_0 _is the total solute concentration difference across the boundary.

## Quantization of diffusion process

Diffusion can be considered as movement of molecules from a higher concentration to a lower concentration. In reference to Eq. (2), the forces acting on the diffused particle are the driving and the friction forces:(11)

In order to construct the Hamiltonian of the Diffused particle we should obtain the potential corresponding to this force. By using the formula [8] (see the appendix):(12)

which enables us to have the potential of a nonconservative force, the potential corresponding to the velocity dependent term which represents the frictional force dissipation effect is(13)

Where (see the appendix)(14)

The driving force is the random force  may be represented as a sequence of impulses between the particle assembly; in the same way we think of pressure that it is just the force per unit area due to a tremendous number of impacts of individual molecules. Hence, we can replace the potential that produces the force of one impulse or one collision *V'*(*x*) by - *δ*(*x'*-*x*) and the entire potential, *V*(*x*), will be written as(15)

Using the identities [9](16)

and(17)

which leads to(18)

Thus, the force *F*(*x'*) is obtained directly(19)

At the same time,  could be written as(20)

this random force may expressed spatially, instead of its time dependence, in the same way as(21)

By making use of Eq.(19)(22)

Thus, we obtain a definition of the random force, that agrees with our assumption and with the fact that ⟨*F(x)*⟩ = 0 of Eq.(2).

The Lagrangian of the Diffused particle is(23)

where(24)

The generalized Euler-Lagrange equation for this problem, reads as [10,11]:(25)

That leads to(26)

which is the classical equation of motion of the diffused particle, Eq.(2).

The canonical momenta are [10,11]:(27)

and,(28)

Making use of the Hamiltonian definition [1,2](29)

the Hamiltonian of the Diffused particles is(30)

Here, *p*_0 _and *p*_1/2 _are the canonical conjugate momenta to *q*_0 _and *q*_1/2 _respectively.

Schrödinger equation reads [1,2](31)

Making use of Eqs. (32 and 33), Schrödinger equation reads as:(32)

Using the method of separation of variables, the relations in the appendix, and defining Ψ as(33)

we find that the time-dependent part is(34)

and has the solution(35)

The other part is:(36)

where *q*_0 _= *x *and *q*_1/2 _= *y*.

Now, let *x *= *uy*. Substituting into Eq. (36), we have(37)

As an approximation, we assume constant values of *u*. This leads to(38)(39)

For *y *≠ *y' *Eq.(39) is reduced to(40)

which has the solution[12,13](41)

*H*_*n *_being Hermite polynomials.

The *y *= *y' *of Eq.(39) will be ignored since the impulse potential effects will be considered in the part of Eq. (37) which will be written as(42)

where we assumed *y *is a constant. Using the identity [9](43)

will reduce Eq.(42) to(44)

which has the solution[9](45)

where *H*(*u *- *u'*) Heaviside step function.

Thus(46)

In terms of *q*_*s(i)*_, Ψ is expressed as(47)

## Application: osmosis

Osmosis is a physical phenomenon that has been extensively studied by scientists in various disciplines of science and engineering. Early researchers studied the mechanism of osmosis through natural materials, and from the 1960s, special attention has been given to osmosis through synthetic materials. Following the progress in membrane science in the last few decades, especially for reverse osmosis applications, the interests in engineered applications of osmosis has been spurred. Osmosis, or as it is currently referred to as forward osmosis, has new applications in separation processes for wastewater treatment, food processing, and seawater/brackish water desalination. Other unique areas of forward osmosis research include pressure-retarded osmosis for generation of electricity from saline and fresh water and implantable osmotic pumps for controlled drug release. This paper provides the state-of-the-art of the physical principles and applications of forward osmosis as well as their strengths and limitations.

**Figure 1:**
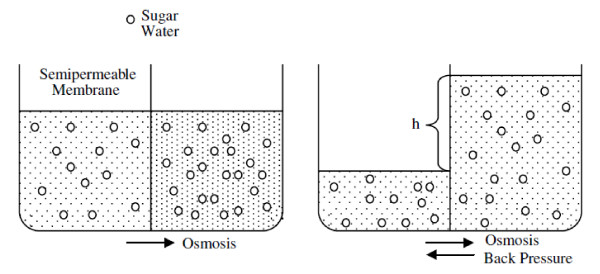
**Osmosis **[6].

*Osmosis *is usually defined as the transport of molecules in a fluid through a semipermeable membrane due to an imbalance in its concentration on either side of the membrane [6]. Osmosis may be by diffusion, but it may also be a bulk flow through pores in a membrane. If a plant cell is put into a concentrated solution of sugar, for example, Fig. 1, the pressure on the right is then, in either case, water moves from a region of high concentration to a region of low concentration greater than the pressure on the left by an amount *hρg*, where *ρ *is the density of the liquid on the right and is called the relative osmotic pressure. The general formula for the osmotic pressure *P *of a solution containing *n *moles of solute per volume *V *of solvent is [6](48)

The net osmotic pressure exerted on a semipermeable membrane separating the two compartments is thus the difference between the osmotic pressures of both compartments.

Making use of the proportionality of pressure and force, Eq. (11) becomes(49)

Where *F*_*p *_is the force per unit area; thus Eq. (22) becomes(50)

Thus, we obtain a definition of the new random force, but with a complete disagreement with our assumption with the fact that ⟨*F(x)*⟩ = 0 of Eq.(2). This disagreement appears from the fact that pressure exerted a net drift force *F*_*p *_on the particles in the direction of osmosis (Fig. 1).

At the same time Eq. (47) looks like(51)

## Discussion

Fraction calculus is very helpful expressing the dissipation, as well as in quantizing nonconservative systems associated with many important physical problems: either where the ordinary quantum-mechanical treatment leads to an incomplete description, such as the energy loss by charged particles when passing through matter; or where it leads to complex nonlinear equations, such as Brownian motion, and diffusion.

By using fractional calculus in physical problems it is possible to create a whole mechanical description of nonconservative systems, including Lagrangian and Hamiltonian mechanics, canonical transformations, Hamilton-Jacobi theory, and quantum mechanics. In this paper, an important physical nonconservative system, which is the diffusion process is treated quantum-mechanically, for the first time using fractional-calculus.

**Figure 2:**
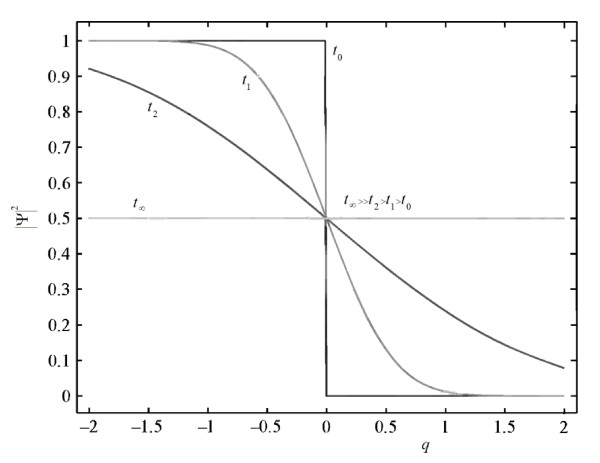
**Probability function**. Probability function, |Ψ|^2 ^of the diffused particle including the random, and frictional forces; the osmosis process manifested very clearly.

Well-known biological process correlated diffusion is studied. Fig. 2 represents the fun probability function, |Ψ|^2 ^of Eq. (51), connected to of the "osmosisized" particle including the drift, and frictional forces; the osmosis process is manifested very clearly, where the confinement of these particles to one region of space gradually leads to a situation in which the particles uniformly fills all the available space in the high concentration, where the new Heaviside step function, *H*_*p*_, is modified due to drift force to show non-step behavior; which agrees totally with the macro-scale view, or the classical-version osmosis.

## Conclusion

Diffusion and the diffusion equation are central topics in both Physics and Mathematics, and their ranges of applicability span from astrophysical dynamics to the diffusion of particles governed by Schrödinger's equation.

The quantization of a system with diffusion process has been carried out according to the theory proposed recently [1,2]. A potential, and a Hamiltonian, corresponding to the random force, and dissipative force, were constructed. The relevant Schrödinger's equation has then been set and solved. The classical equation of motion, connected to diffused particle, could be obtained easily from the fractional Lagrangian. The random and frictional forces were plotted; the diffusion process manifested very clearly. The next step could be to study problems such as the correlation functions, transport equation, chemical potential, entropy, etc., on a quantum-mechanical basis.

An application of the developed mathematical method to the analysis of diffusion in a biological medium, osmosis, is carried out. Schrödinger's equation is solved. The plot of the probability function represents clearly the dissipative and drift forces and hence the osmosis, which shows the same macro-scale view of the osmosis.

## Appendix: Fractional calculus

The fractional integral of a function *f*(*t*) is defined as [14,15](A.1)

where *J*^α ^represents the fractional integral operator of order *α*, and R^+ ^represents the set of positive real numbers.

If we introduce the positive integer *m *such that *m *-1 <*α *≤ *m *the fractional derivative of order *α *> 0 may be defined as(A.2)

*D*^*a *^being the fractional deferential operator of order *a *Equation (2) may be rewritten using Eq. (1) as follows:(A.3)

Here, we formulate the problem in terms of the left fractional derivative the left Riemann-Liouville fractional derivatives, which are defined in Eqs. (A.1, A.2). Most of the left fractional operations also hold for the right ones. For the left operations *f*(*t*) must vanish for *t *<*a *while *f*(*t*) = 0 for *t *>*b *for the right operation. Thus, the left operations are causal. Conversely, the right operations are anti-causal [16]. From the physical point of view, when we differentiate with respect to time, the right differentiation represents an operation performed on the future state of the process *f*(*t*) [17].

Fractional integral and differential operators have the following properties [14,15]:

For *I*, the identity operator:(A.4)

but the inverse application of the two operators is not necessarily true.

For *n *> 0, *J*^*n *^and *D*^*n *^are linear operators, i.e.,(A.5)(A.6)

For a constant *c*, *J*^*n *^and *D*^*n *^are homogeneous operators, i.e.,(A.7)(A.8)

For *α*, *β *> 0, *J*^*n *^obeys the additive index law, but not necessarily *D*^*n*^, i.e.,(A.9)(A.10)

Of special importance are the fractional integrals and fractional derivatives of the function (*t *- *a*)^*β*^, which are given by(A.11)

For *α *= 1/2 this equation is called semi-derivative; for *α *= - 1/2 it is called semi-integral.

## Competing interests

The authors declare that they have no competing interests.

## Authors' contributions

**AWA**: 1) has made extensive contributions to conception and design of the manuscript, and analysis and interpretation of data; 2) has been involved in drafting the manuscript; and 3) has set the final approval of the version to be published. He has participated sufficiently in the work to take public responsibility for appropriate portions of the content.

**HR**: 1) has been involved in revising the manuscript critically in its mathematical content. He has participated sufficiently in the work to take public responsibility for appropriate portions of the content.

All authors read and approved the final manuscript.

## Authors' information

**Abdul-Wali Ajlouni (Jordan) **is the chair of the Applied Physics Department at Tafila Technical University, Jordan. Prior to this post, he served as an Associate Dean of the Faculty of Science, and as an assistant Professor. Before joining TTU, Dr. Ajlouni was a researcher on radiation safety for the Ministry of Energy. He has received training on radiation protection and radiological emergency preparedness in Jordan, Turkey, the United States, Czechoslovakia, Egypt, Iran, and Syria. In addition, he has attended numerous conferences on nuclear energy, and Applied mathematics around the world. Dr. Ajlouni has published extensively on the two subjects, with titles such as: "Mathematical Model for Dispersion of Nuclear Pollutants in Jordan Atmosphere," and "Quantization of Brownian Motion." Dr. Ajlouni received a SCOPUS Scientific Research and the International Conference on Mathematics and Information Security (ICAMIS2009) awards in 2009. He holds a B.Sc. in Physics from Yarmouk University, Jordan, an M.S. in Nuclear Physics from Mustansyria University, Iraq, and a Ph.D. in Mathematical/Radiation Physics from the University of Jordan.
